# Room Temperature Co-Precipitation Synthesis of Magnetite Nanoparticles in a Large pH Window with Different Bases

**DOI:** 10.3390/ma6125549

**Published:** 2013-11-28

**Authors:** Maria Cristina Mascolo, Yongbing Pei, Terry A. Ring

**Affiliations:** 1 Department of Chemical Engineering, University of Utah, Salt Lake City, UT 84112, USA; E-Mails: yongbingpei@gmail.com (Y.P.); t.ring@utah.edu (T.A.R.); 2 Laboratory of Materials, Department of Civil and Mechanical Engineering, University of Cassino and Southern Latium, G. Di Biasio 43 Street, 03043 Cassino (FR), Italy; 3 Key Laboratory of Organosilicon Chemistry and Material Technology of Ministry of Education, Hangzhou Normal University, Hangzhou 310036, China

**Keywords:** magnetite nanoparticles, co-precipitation, room temperature, superparamagnetism, aggregation, mesoporous structure

## Abstract

Magnetite nanoparticles (Fe_3_O_4_) represent the most promising materials in medical applications. To favor high-drug or enzyme loading on the nanoparticles, they are incorporated into mesoporous materials to form a hybrid support with the consequent reduction of magnetization saturation. The direct synthesis of mesoporous structures appears to be of interest. To this end, magnetite nanoparticles have been synthesized using a one pot co-precipitation reaction at room temperature in the presence of different bases, such as NaOH, KOH or (C_2_H_5_)_4_NOH. Magnetite shows characteristics of superparamagnetism at room temperature and a saturation magnetization (Ms) value depending on both the crystal size and the degree of agglomeration of individual nanoparticles. Such agglomeration appears to be responsible for the formation of mesoporous structures, which are affected by the pH, the nature of alkali, the slow or fast addition of alkaline solution and the drying modality of synthesized powders.

## 1. Introduction

Magnetite nanoparticles have applications in several areas, such as biomedical [1,2,3], target drug delivery [4], tumor and cancer diagnosis and treatment [5] and as a magnetic resonance imaging (MRI) contrasting agent [6,7]. These medical applications require superparamagnetic magnetite with particle sizes smaller than 20 nm [8].

Magnetite adsorbs oxygen to form maghemite, which, in turn, loses its susceptibility with time [9]. On the other hand, the nanosized particles form less stable systems from the colloidal point of view, determining their agglomeration. In the case of magnetic fluids, to prevent agglomeration, the surface coating of a particle is essential for having stable colloidal dispersion in a wide pH range [10,11]. In addition, the adsorption layer can also enhance the resistance against oxidation of magnetite into maghemite (γ-F_2_O_3_).

The applications of magnetite nanoparticles depend on the preparation method, which, in turn, influences particle size and shape, size distribution, agglomeration and the surface chemistry of the material.

The magnetite can be synthesized by various methods, including: ultrasound irradiation [12], sol-gel [13], thermal decomposition [8,14,15,16,17,18] and co-precipitation [19,20,21,22,23,24,25]. Thermal decomposition and co-precipitation are most commonly used. The thermal decomposition route relies on the pyrolysis of organic precursors of iron, such as Fe(CO)_5_ [14,15] and Fe(acac)_3_ [8,16,17,18]. However, Fe(CO)_5_ and Fe(acac)_3_ are toxic and may limit the application of magnetite for medical applications. Recently, a one pot synthesis of magnetite by thermal decomposition of [Fe(CON_2_H_4_)_6_](NO_3_)_3_ [26] was reported. Co-precipitations based on the hydrolysis of a mixture of Fe^2+^ and Fe^3+^ ions are used to fix the A to B molar ratio in the inverse spinel structure. Generally, the reaction is performed under an inert (N_2_ or Ar) atmosphere using degassed solutions to avoid uncontrollable oxidation of Fe^2+^ into Fe^3+^ [24]. In this method, Fe^2+^ and Fe^3+^ ions are generally precipitated in alkaline solutions, such as ammonium hydroxide, potassium hydroxide or sodium hydroxide. In most cases, the syntheses are performed at 70–80 °C [25] or higher temperatures. The effects of mixing methods [20], stirring rate [27], digestion time [28], initial pH [29] and the presence or absence of a magnetic field [19,30] on particle size, morphology and resulting magnetic properties were also discussed. Co-precipitation methods performed under various precipitation conditions were also reported. For example, when only Fe^2+^ was used for precipitation, then H_2_O_2_ [19,20] or NaNO_2_ [21] were adopted to partially oxidize Fe^2+^ into Fe^3+^ in the precipitated product. When only Fe^3+^ was used for precipitation, then Na_2_SO_3_ [22] partially reduces ferric to ferrous ion in the precipitation product. Some co-precipitation methods are performed in the presence of polymers [23], including: polyvinyl alcohol (PVA) and dextran to prevent both agglomeration and/or oxidation of the nanoparticles. All these co-precipitation methods are comparatively complex and require strict control of precipitation conditions.

The magnetic properties of magnetite nanoparticles strongly depended on the synthesis route. In particular, size determines the magnetite properties [31,32]. The saturation magnetization can be most likely affected by several features, such as the spin disorder layer [33], that increase with a decrease in crystallite size.

Other factors include the finite size effect, the incomplete crystallization of magnetite, irregular morphologies of magnetite particles [34] and the magnetostatic interaction responsible for the agglomeration of magnetite particles [35].

The synthesis of mesoporous structures of magnetite appears of interest, because the presence of mesopores favors high drug or enzyme loading of magnetite for medical application. To this end, a cheap coprecipitation method of synthesis is proposed. The main objective of this work concerns the formation of mesoporous structures by agglomeration of magnetite nanoparticles.

Alkaline solutions containing stoichiometric or excesses of NaOH, KOH or (C_2_H_5_)_4_NOH, respectively, were used. Because many of the coprecipitation methods are performed at elevated temperatures [25], to evaluate the effect of temperature on the synthesis magnetite, a computer software, developed by OLI Systems, Inc. (Morris Plains, NJ, USA), was also used.

## 2. Experimental

### 2.1. Chemicals and Equipment

The chemicals used, FeCl_3_·6H_2_O, FeCl_2_·4H_2_O, NaOH, KOH and (C_2_H_5_)_4_NOH, were purchased from Sigma-Aldrich and used without further purification. To avoid the formation of both maghemite, γ-Fe_2_O_3_, and hematite, α-Fe_2_O_3_, all the syntheses were performed under oxygen-free conditions.

Two series of samples have been prepared: the first one by slow addition, 1.0 mL/min, of the alkaline solution into the reaction solution, whereas the second series was obtained by a fast addition of the alkaline solution to the reaction solution. A precision pump operating at a flow rate of 1.0 ± 0.1 mL/min was used for the addition of reactants to the reaction vessel. In order to evaluate the effect of both temperature and pH on the magnetite synthesis, the OLI (SLA) software, was used. In this software, solution equilibria account for all solution complexes and the solubility of various solids, simultaneously. Table 1 gives a list of solution complexes and solids phases. The solutions are non-ideal, with activity coefficients being calculated for each species in solution using extended the Debye–Hückel theory.

The solution conditions used for this analysis are 0.1 mol/L FeCl_3_, 0.05 mol/L FeCl_2_ in deionized water and various NaOH concentrations to give various pH values at equilibrium. Solution equilibria are calculated for different temperatures from 0 to 100 °C at one atmosphere total pressure.

**Table 1:** Solution complexes and solid phases in equilibria calculations.

Solid Phases	Solution Complex Species		
Fe(OH)_2_	FeCl_2_	OH^−^	[FeCl_2_]^+^
Fe(OH)_3_	FeCl_3_	Fe^2+^	[Fe(OH)_2_]^+^
NaCl	HCl	Fe^3+^	[FeCl]^2+^
FeCl_2_·4H_2_O	Aqueous Fe(OH)_3_ and Fe(OH)_2_ precipitation precursors	[FeCl]^+^	[Fe(OH)]^2+^
FeCl_2_	Cl^−^	[FeOH]^+^	[FeCl_4_]^−^
NaOH·H_2_O	[Fe_2_(OH)_2_]^4+^	[Fe(OH)_4_]^2−^	[Fe(OH)_4_]^−^
FeCl_3_·6H_2_O	H^+^	[Fe(OH)_3_]^−^	Na^+^

### 2.2. Synthesis

Fe_3_O_4_ was synthesized in alkaline conditions using different bases, BOH, with B = Na^+^, K^+^ or (C_2_H_5_)_4_N^+^, to complete the following overall reaction:

2FeCl_3_ + FeCl_2_ + 8BOH → Fe_3_O_4_ (s) + 4H_2_O + 8BCl (1)

To investigate the effect of pH value on the properties of magnetite nanoparticles, the experiments were carried out at various BOH amounts. During the reaction, the reaction flask was equipped with a pH meter to measure the final pH value. The experimental parameters for the synthesis of magnetite nano-particles are summarized in Table 2. The coprecipitation of iron ions has been performed either with stoichiometric or with an excesses of BOH solution, adopting a slow (S) or a fast (F) addition of the precipitating alkaline solution.

In a typical experiment (S1 sample), 0.01 mol FeCl_2_∙4H_2_O and 0.02 mol FeCl_3_∙6H_2_O were dissolved in 100 mL degassed deionized water and added into a 250 mL three-neck flask, which was immersed in a room temperature (25 °C) water bath. Point-zero-eight moles of NaOH were dissolved in 100 mL degassed deionized water. The reaction solution was mechanically stirred at 500 rpm while nitrogen gas flowed into the flask. After charging of the reactor with NaOH was completed, stirring continued for 3 h, after which the final pH was recorded. After this reaction time, the solution was decanted, allowing the particles to be washed with degassed deionized water. This procedure was repeated three times, and then, the particles were separated by a permanent magnet and dried in a vacuum desiccator.

**Table 2:** Experimental parameters.

Sample	FeCl_3_·6H_2_O (mol)	FeCl_2_·4H_2_O (mol)	BOH (mol)	Final pH
S1	0.02	0.01	0.08 (NaOH) ^**^	10.34
S2	0.02	0.01	0.085 (NaOH)	11.94
S3	0.02	0.01	0.09 (NaOH)	12.08
S4	0.02	0.01	0.095 (NaOH)	12.20
S5	0.02	0.01	0.1 (NaOH)	12.60
F1_Na_	0.02	0.01	0.08 (NaOH) ^**^	9.4
F3_Na_	0.02	0.01	0.09 (NaOH)	10.7
F5_Na_	0.02	0.01	0.1 (NaOH)	11.8
F3_K_	0.02	0.01	0.09 (KOH)	–
F3_TEA_	0.02	0.01	0.09 (TEAOH)	–
* F3_TEA_	0.02	0.01	0.09 (TEAOH)	–

^*^ Fast and reverse addition of solution containing ferric and ferrous ions into the TEAOH-containing solution; ^**^ stoichiometric amount of base.

### 2.3. Characterization

In order to verify an eventual oxidation of magnetite by the formation of secondary phases, such as maghemite, γ-Fe_2_O_3_, and/or hematite, α-Fe_2_O_3_, the as-synthesized products were analyzed in the Fe^2+^/Fe^3+^ molar ratio. The samples were dissolved in H_2_SO_4_ 3M under O_2_-free conditions. The Fe^2+^ content was analyzed by permanganometric determination, while the total iron (Fe^2+^ + Fe^3+^) was determined by atomic absorption spectrophotometry. The quantity of crystalline phases was determined by Rietveld quantitative analysis using an X-ray diffractometer (PA Nalytical B.V. Almelo, the Netherlands). Data collection was performed using Cu-K_α_ (λ = 0.154060 nm) radiation (step time, 10 s; step size, 0.05°; 2θ angular range = 5°–95°).

The specific surface area of the powders was measured by the Brunauer–Emmett–Teller (BET) method and the pore size distribution of the powders by the adsorption-desorption isotherm utilizing nitrogen as the adsorbate after drying at 80 °C for 12 h (Gemini 2375, Micromeritics Instrument Inc., Norcross, GA, USA).The magnetite nanoparticle morphology was investigated by scanning electron microscopy (SEM) (FEI Quanta 600 FEG, Hillsboro, OR, USA) with a field emission electron gun operated at 25 kV. The transmission electron microscopy (TEM) study was carried out on an electron microscopy instrument (FEI Tecnai G12 Spirit Twin, Hillsboro, OR, USA). The powders for TEM were dispersed in ethanol; the suspension was then dropped on carbon-copper grids.

The magnetic properties of the particles were measured with a Physical Properties Measurement System magnetometer at 298 K (Quantum Design, San Diego, CA, USA). The magnetization measurement was taken from the −10 kOe to 10 kOe field. From the magnetization *versus* applied field curve, the saturation magnetization (Ms) was measured.

## 3. Results

**Figure 1:**
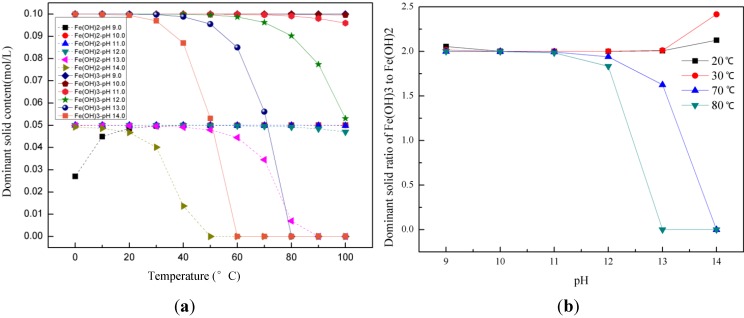
(**a**) Dominant solids (Fe(OH)_2_ and Fe(OH)_3_) concentration (mol/L) as a function of temperature at various pH values. Other solids are not precipitated at these conditions; and (**b**) Dominant solids ratio of Fe(OH)_3_ to Fe(OH)_2_ at room temperature (20–30 °C) and high temperature (70–80 °C) at various pH values.

Figure 1a shows the solid concentration in equilibrium with the solution as a function of temperature for various pH conditions, which are equivalent to various amounts of NaOH added during co-precipitation. Here, we see that a stoichiometric ratio of Fe^3+^ to Fe^2+^ equal to 2:1 in the solid phase is best achieved at room temperatures, which give the largest window of pH values, from 10.0 to 13.0, where a near perfect 2:1 stoichiometric solid may be produced. At higher temperatures 70–80 °C, the product precipitated from a 2:1 stoichiometric solution is not stoichiometric, except in a narrow pH window. The dominant solids of Fe^3+^ and Fe^2+^ ions start to deviate from 0.1 and 0.05 mol/L at pH values greater than 12.0 or 13.0, depending upon temperature. Figure 1b shows the solids ratio of Fe(OH)_3_ to Fe(OH)_2_. We see that at 20–30 °C, this ratio stays perfect close to 2.0 in the pH window from 10.0 to 13.0. At higher temperatures 70–80 °C, a 2:1 stoichiometric solution produces a 2:1 stoichiometric solid at pH values less than 12.0, and this ratio starts to deviate from 2.0 at pH values greater than 12.0. This is contrary to the method of increasing pH (OH^−^ concentration) to high values in order to completely precipitate the iron species as hydroxides that is used by experimentalists. These results justify the reason for which all the syntheses have been performed at room temperature.

The co-precipitation method using Fe^2+^ and Fe^3+^ ions reacting in alkaline conditions has been extensively investigated [24,27,28,29,30], and the following reactions are proposed for the mechanism of magnetite formation:

Fe^3+^ + 3OH^−^ = Fe(OH)_3_ (s) (2)

Fe(OH)_3_ (s) = FeOOH (s) + H_2_O (3)

Fe^2+^ + 2OH^−^ = Fe(OH)_2_ (s) (4)

2FeOOH (s) + Fe(OH)_2_ (s) = Fe_3_O_4_ (s) + 2H_2_O. (5)

Giving an overall reaction:

2Fe^3+^ + Fe^2+^ + 8OH^−^ = 2Fe(OH)_3_Fe(OH)_2_ (s) → Fe_3_O_4_ (s) + 4H_2_O (6)

First, the ferric and ferrous hydroxides are precipitated. These reactions are very fast. Second, the ferric hydroxide decomposes to FeOOH, due to the low water activity of the resulting NaCl solution in a slower reaction. Finally, a solid state reaction between FeOOH and Fe(OH)_2_ takes place, due to the low water activity of the solution, which produces magnetite. This solid state reaction takes place between 10 and 30 min at room temperature. The overall reaction mechanism is a dynamic equilibrium equation in which the concentration and size of Fe_3_O_4_ nanoparticles are influenced by [Fe^3+^], [Fe^2+^] and [OH^−^], as well as the water activity of the solution. We design ferric to ferrous molar ratio as 2:1, which is the exact stoichiometry for magnetite, in order to produce high purity magnetite nanoparticles. The final [OH^−^] concentration, which is related to the pH and base amount, is known to control the nucleation and growth of the magnetite nanoparticles and can influence the magnetite properties, e.g., particle size and saturation magnetization (Ms). In this study, the room temperature syntheses of magnetite, obtained at various pH values by varying the nature of the base and its amount, are reported in Table 2.

### 3.1. Elemental Analysis

The individual Fe^2+^ content of the products was obtained by permanganometric determination, while the amount of the individual Fe^3+^ was calculated from the difference between the total iron and the Fe^2+^ content. The experimental Fe^2+^/Fe^3+^ molar ratio being of the order of 0.45 (Table 3) with respect to the theoretical ratio of 0.5 proves that the synthesized magnetite products are pure enough.

**Table 3:** Fe^2+^/Fe^3+^ molar ratio determined by elemental analysis and quantitative crystalline phases determined by the Rietveld method for the synthesized products.

Sample	Fe^2+^/Fe^3+^ Molar ratio	Magnetite (wt %)	Maghemite (wt %)	Hematite (wt %)
S1	0.48	98.0	1.5	0.5
S2	0.47	96.7	2.9	0.4
S3	0.45	95.5	4.2	0.3
S4	0.46	96.1	3.3	0.6
S5	0.43	93.9	5.8	0.4
F1_Na_	0.49	98.2	1.6	0.2
F3_Na_	0.44	94.0	5.5	0.5
F5_Na_	0.42	91.1	8.7	0.2
F3_K_	0.43	92.3	6.3	0.4
F3_TEA_	0.41	90.3	9.4	0.3

### 3.2. XRD Pattern

An estimation of magnetite content for the products, determined by the Rietveld XRD quantification, are also reported in Table 3. A good agreement between the data of elemental analysis and those of quantitative phase analysis by the Rietveld method results.

**Figure 2:**
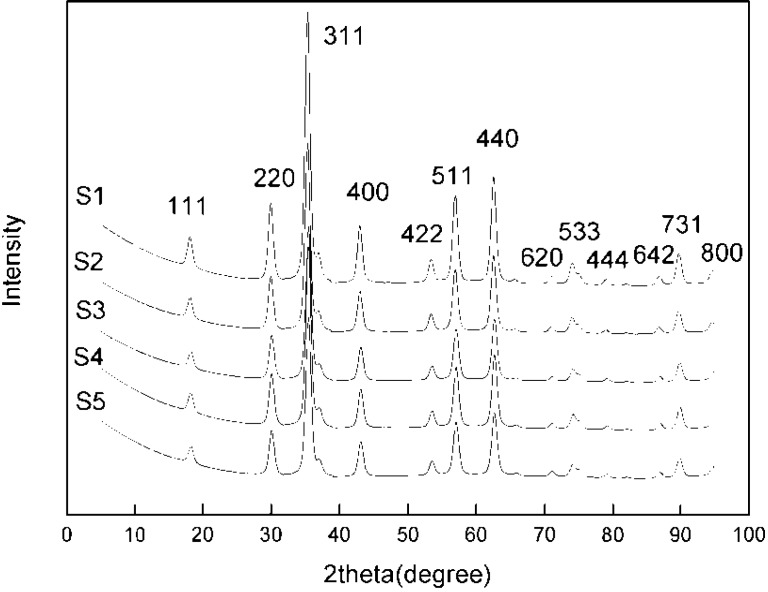
X-ray diffraction of magnetite nanoparticles synthesized at various NaOH amounts.

Figure 2 shows the XRD patterns of magnetite particles synthesized by slow addition at various NaOH amounts and pH values. The XRD diffraction peaks correspond well to magnetite Fe_3_O_4_ (JCPDS file, No. 00-011-0614). The products synthesized by fast addition also show XRD patterns typical of magnetite particles.

An estimation of the magnetite nanoparticles size has been performed from the Scherrer formula:

(7) $$D=\frac{\text{K}\lambda }{B\text{cos}\theta }$$

where λ is the X-ray wavelength (0.15406 nm), B is the full width at half maximum (FWHM); θ is the corresponding Bragg angle; and K is the shape parameter, which is 0.89 for magnetite. Taking the highest intensity peak, namely the (311) plane, at 2θ = 35.4°, and the half maximum intensity width of the peak after accounting for instrument broadening, the calculated particle sizes are 11.5, 11.2, 11.0, 10.9 and 10.7 nm for samples S1, S2, S3, S4 and S5, respectively (the second column of Table 3). As the NaOH amount and pH increases, the nanoparticle size decreases, albeit a small amount. At higher pH, supersaturation during co-precipitation is higher, promoting nucleation over growth, thus giving smaller particle sizes. By using the co-precipitation method over this wide range of pH values at room temperature, it is easy to prepare magnetite nanoparticles with an approximate size of 11.0 nm.

**Figure 3:**
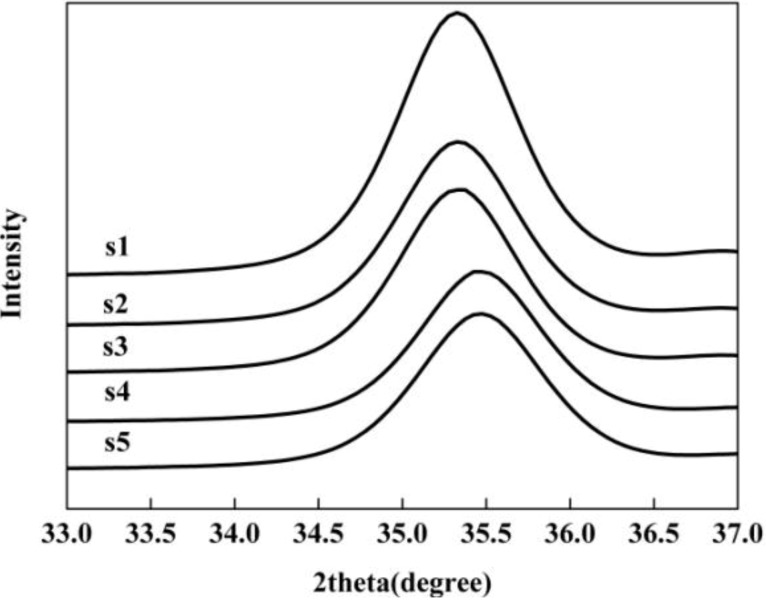
(311) peaks of the magnetite nanoparticles in expanded 2θ scale.

Note that in the XRD patterns, the peak positions shift slightly forward to larger 2θ values with increasing pH. The (311) XRD peak of the products as a magnified representation clearly shows the peak shift, indicating that the lattice parameter contracts as the particle size decreases (Figure 3). This phenomenon has also been observed and reported in previous works [28,31].

The fast addition of the stoichiometricor of the more concentrated NaOH solution to the reaction solution determines the formation of magnetite nanoparticles smaller in sizes,10.2 and 8.2 nm for F1_Na_ and F5_Na_ samples, respectively, in comparison to those of the corresponding magnetite obtained by slow addition of the base. The fast addition favors the continuous nucleation with respect to growth, thus enabling the formation of small particles. The nature of the precipitating base also affects the particle sizes. Smaller magnetite nanoparticles of 7.1 and 6.5 nm result for F3_K_ and F3_TEA_ samples synthesized by fast addition of KOH and TEAOH, respectively. The sample * F3_TEA_, synthesized with a reverse modality by fast addition of ferrous and ferric ions into TEAOH solution, shows magnetite nanoparticles of 6.4 nm determined by TEM, a size very similar to that of the F3_TEA_ sample synthesized by fast addition of TEAOH solution into the iron chloride-containing solution.

Although the syntheses were performed under O_2_-free conditions, a certain oxidation of magnetite into maghemite results. By comparing the maghemite content detected in the synthesized products (Table 3) with the corresponding crystal sizes shown in Table 4, it can be seen that the smaller the particle sizes of magnetite, the higher the maghemite content. These results agree with the higher reactivity of magnetite characterized by a crystal smaller in size.

**Table 4:** Properties of magnetite samples. Ms, saturation magnetization.

Sample	Size (nm)		Specific surface area (m^2^/g)	Specific surface area (m^2^/g)	Interface area (m^2^/g)	Ms(emu/g)
	XRD	BET	XRD	BET	(S_XRD_-S_BET_)/2	
S1	11.5	12.9	100.7	89.4	5.6	75.3
S2	11.2	12.4	103.4	93.3	5.1	71.6
S3	11.0	12.0	105.3	97.1	4.1	69.8
S4	10.9	11.8	106.3	97.8	4.3	69.4
S5	10.7	11.6	108.3	99.4	4.5	68.3
F1_Na_	10.2	11.8	113.6	98.1	7.7	64.8
F3_Na_	9.1	10.0	127.0	109.0	9.0	58.1
F5_Na_	8. 2	8.9	141.3	130.0	5.6	54.1
F3_K_	7.1	7.6	163.1	152.4	5.3	53.6
F3_TEA_	6.5	6.9	178.2	167.9	5.1	52.3

### 3.3. BET Measurement

**Figure 4:**
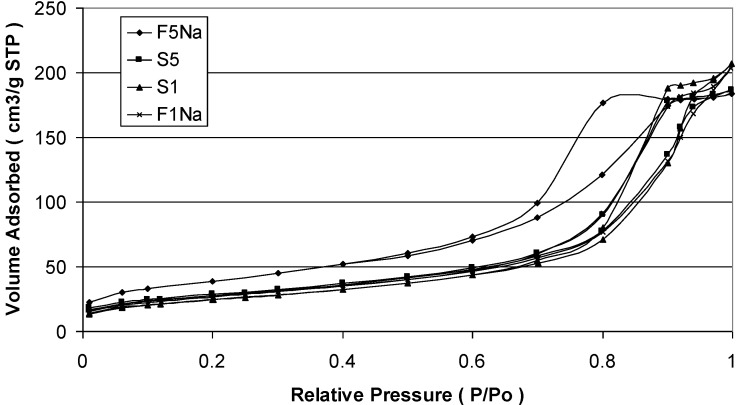
N_2_ adsorption-desorption isotherms of S1, S5, F1_Na_ and F5_Na_ samples.

Table 4 shows also the specific surface area measured by the BET method and saturation magnetization (Ms) of the magnetite nanoparticles synthesized at various conditions. Here, the listed particle size is calculated either from XRD or from BET measurement adopting the particles diameter, *D*, given by:

(8) $$D=\frac{6}{S_{\text{sp}}\rho_{\text{a}}}$$

where *S*_sp_ is the specific surface area of the sample and ρ_a_ is the density 5.18 g/cm^3^. We can see that, when the pH increases, the specific surface area increases and particle size decreases. The sizes calculated from BET measurements, compared with the size from XRD, are larger. Such a difference in size for the magnetite samples can be explained with the agglomeration of the nanosized particles, so determining lower surface area and, consequently, an apparent larger particle size. Such agglomeration has been evaluated determining the interface area among the particles of magnetite adopting the formula (*S*_XRD_ – *S*_BET_)/2, where S_XRD_ is the surface area calculated from crystal size measured with the Sherrer formula, whereas S_BET_ is the surface area determined with the BET method. The agglomeration of the nanosized particles of magnetite is confirmed by the N_2_ adsorption-desorption isotherms shown in Figure 4 for the samples S1, S5, F1_Na_ and F5_Na_, respectively. Each product is characterized, in fact, by a clear hysteresis loop, which is typical of mesoporous products as a consequence of the agglomeration.

**Figure 5:**
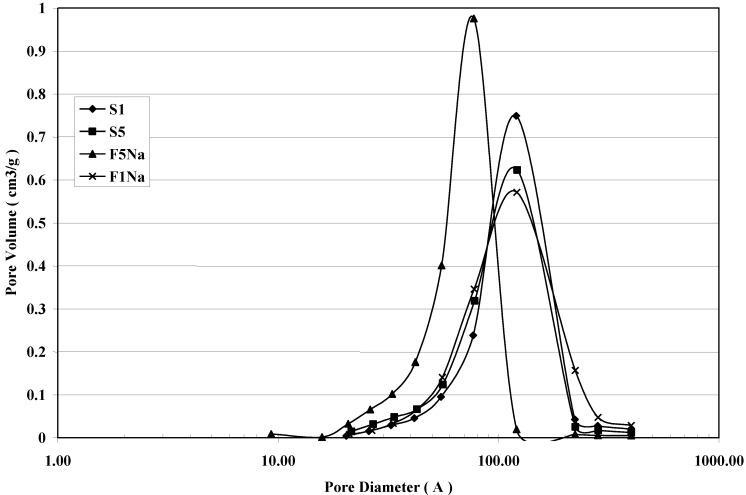
BJH desorption pore size distribution curves for S1, S5, F1_Na_ and F5_Na_ samples.

The corresponding Barret–Joiner–Halenda (BJH) pore size distribution determined from the desorption branch isotherm are shown in Figure 5. A pore size distribution with pores changing in the mesopore range is the result for all the samples. The maximum of the pore size is affected by the particle size of magnetite. The smaller the particle size of magnetite, the lower is the maximum of the pore size results. The fast addition with an excess of the NaOH solution gives a magnetite product with the maximum shifted to smaller values of mesopores. It is also evident that a broader pore size distribution characterizes the products synthesized by the slow addition of the basic solution in comparison to that of the products obtained by the fast addition of the precipitating base.

### 3.4. SEM and TEM Images

**Figure 6:**
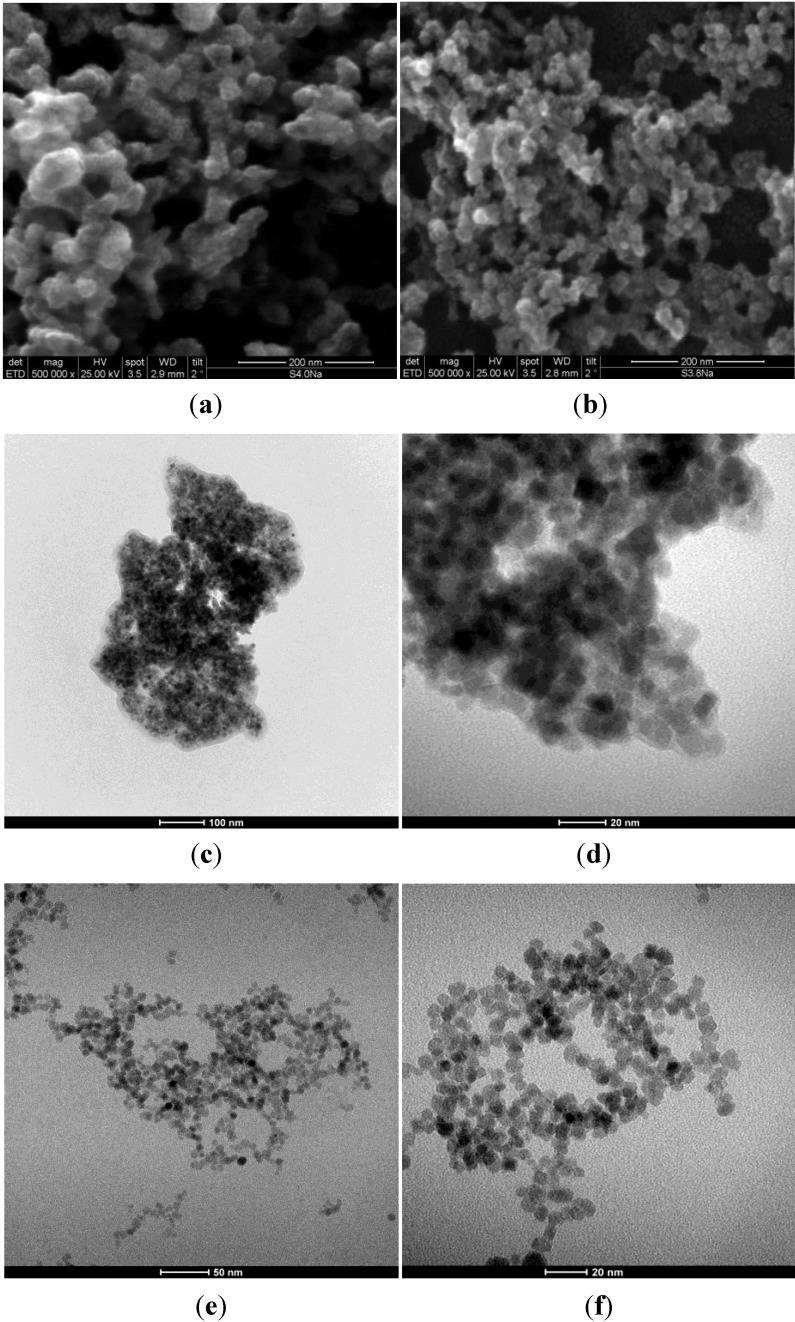
SEM images of (**a**) S4; (**b**) S5 samples; and TEM images of (**c**,**d**) F3_Na_; and (**e**,**f**) * F3_TEA_.

Figure 6 shows SEM images of S4 and S5 samples and TEM images of F3_Na_ and * F3_TEA_ samples, respectively. SEM images show that the samples consist of particles with a nearly spherical shape. They are approximately 11.0 nm in size, indicating that homogeneous magnetite nanoparticles can be synthesized in a large pH window at room temperature.

The magnetite particles are more or less agglomerated, so determining, in the absence of a surfactant, the formation of aggregates with a broad range of mesopores, as can be seen in the TEM images of Figure 6c,d. The TEM images of * F3_TEA_ in Figure 6e,f shows single magnetite particles, due to their very poor agglomeration. Such findings justify the different behavior of this sample in terms of the stability suspension of magnetic particles in the liquid medium.

The * F3_TEA_ sample results in a perfect colloidal suspension, due to the absence of any particles edimentation after several months of aging, whereas for all the other samples, the sedimentation of aggregates results in more or less short times of aging.

This behavior can be related to two concomitant effects of the basic solution of the TEAOH. The magnetic particles synthesized in the presence of TEAOH are very small in size, so that their thermal energy is large enough to overcome the energy of the magnetic interactions among the magnetite nanoparticles [32]. The OH^−^ ions of the basic TEAOH-containing solution are adsorbed onto the magnetite particles, determining a negative charge on each particle. As a surfactant, the positively-charged tetraethylammonium ions form a shell around each magnetite particle, so raising the energy required for the particles to agglomerate, stabilizing the corresponding colloidal suspension. For the F3_TEA_ sample, obtained by fast addition of the TEAOH solution into iron chloride-containing solution, the sedimentation of magnetite particles occurs. Such a difference might be related to the more basic medium in the first step of the reaction when iron chlorides are rapidly added to TEAOH.

### 3.5. Magnetic Properties

**Figure 7:**
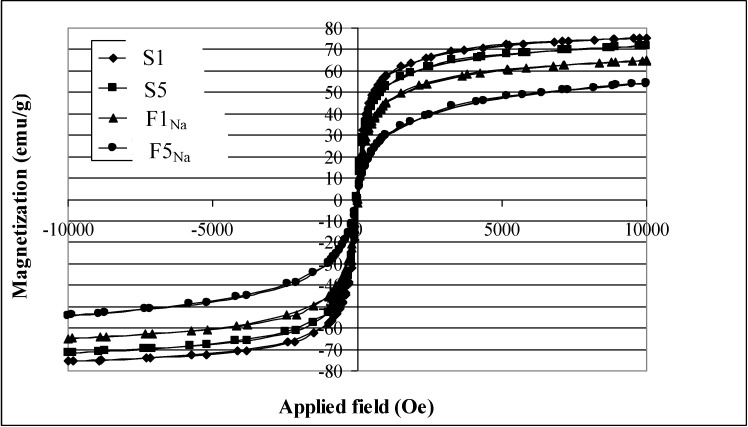
Magnetization curves for S1, S2, F1_Na_ and F5_Na_ samples.

The magnetization curve for the synthesized magnetite nanoparticles reported in Figure 7 does not show any hysteresis behavior for any of the samples and exhibits immeasurable values of coercivity field and remnant magnetization.

This confirms that the synthesized particles exhibit superparamagnetic properties at room temperature. This is in good agreement with a previous study: that magnetite nanoparticles exhibit superparamagnetic properties when they are smaller than the critical size of the magnetic domain size [36,37,38].

Table 4 shows the saturation magnetization (Ms) of all the synthesized samples. We can see that Ms values decrease as the magnetite particle size decreases. This effect has been reported, in great number, previously [26,28,29,31].

## 4. Discussion

The formation of crystalline magnetite from solution by nucleation and growth appears to proceed through rapid agglomeration of nanometric primary particles [39]. Such agglomeration determines branched networks, which, in turn, become denser with the formation of crystalline spheroidal nanoparticles higher in size. The final size of individual particles is affected by several parameters, such as: the pH of solution, reaction temperature, concentration of precursors and slow or fast mixing of reagents [40].

A further agglomeration of individual nanoparticles determines the formation of aggregates higher in size. This last agglomeration appears to be responsible for the formation of mesopores, as can be seen in Figure 4 and Figure 5. Their formation could be attributed to the magnetostatic interaction of magnetite particles. It is not to be excluded that the particles agglomerate during magnetic separation and/or during washing/drying of the nanopowders.

**Figure 8:**
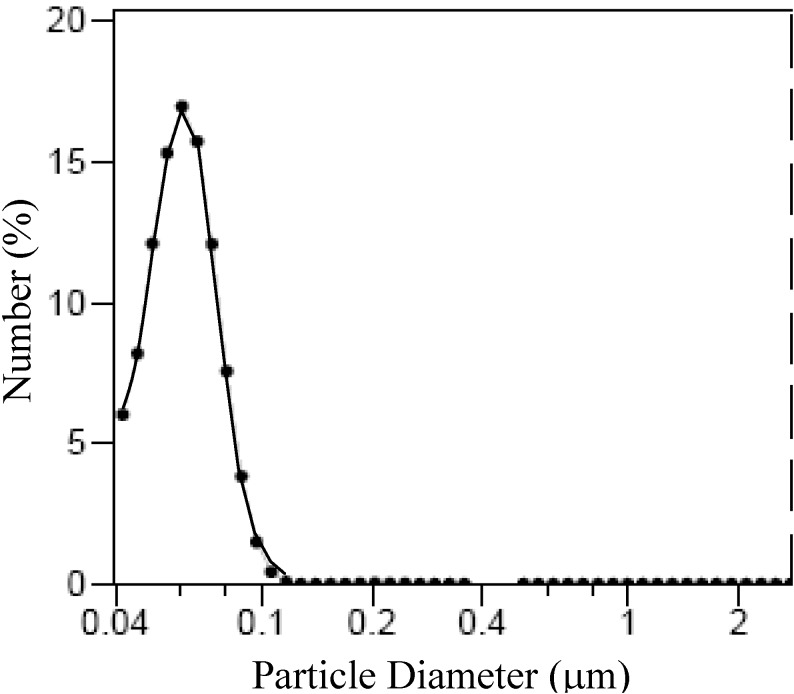
Size distribution of magnetite aggregates determined by laser light scattering (LS).

It must be outlined that with the exclusion of the sample, F^*^_3TEA_, all the synthesized samples exhibit a distribution of the aggregates size (Figure 8) with a median size of 63 ± 25 nm, as determined by laser light scattering (LS). The formation of these aggregates could be mainly attributed to magnetostatic interaction [35].

The aggregates sizes observed by TEM resulted in an order higher size than those detected by LS. Such differences could be attributed to the agglomeration following the magnetic separation and/or washing-drying of the nanoparticles. On the other hand, an increase of interface area with the decrease of particle size must be expected. The resulting interface area for the sample F_3Na_, F_3K_ and F_3TEA_, synthesized with the same concentration of base, contrasts the above sentence. Nevertheless, the particle sizes of magnetite decrease according to the following sequence of counter cations of base: Na^+^ > K^+^ > N(C_2_H_4_)^+^_4_; the interface area decreases, in fact, with the same sequence as reported in Table 4. Such a discrepancy could be attributed to the effect of steric hindrance connected to the cations bigger in size, which hamper the agglomeration among the individual nanoparticles.

Recently, a new strategy to perform bioseparation and diagnostic target isolation under continuous flow processing conditions has been described [41]. Reversible aggregation-disaggregation of magnetite nanoparticles under the action of a pH-responsive polymer coating has been exploited. To this end, the degree of agglomeration of magnetite nanoparticles appears to be of great interest.

**Figure 9:**
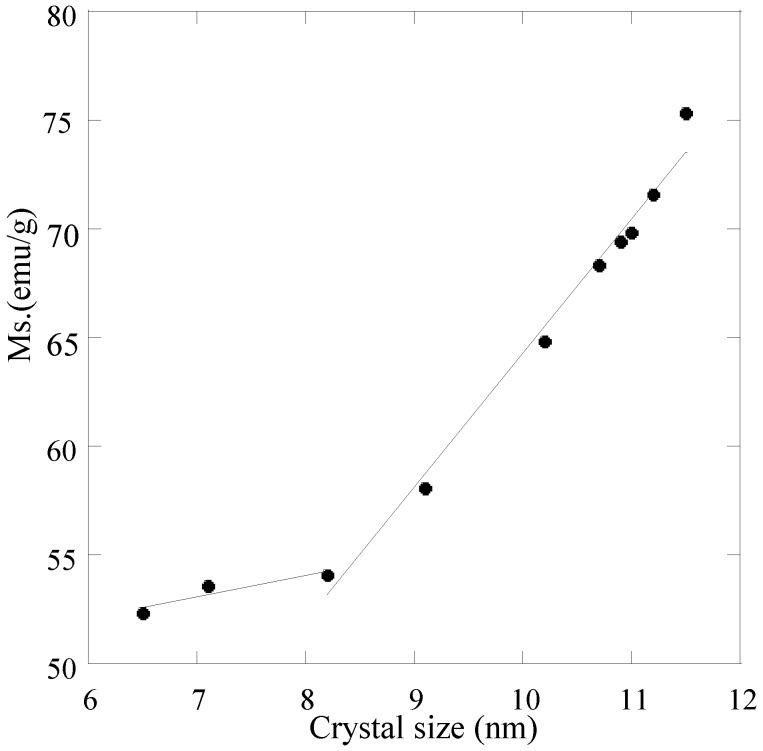
Saturation magnetization (Ms) as a function of magnetite particles size.

**Figure 10:**
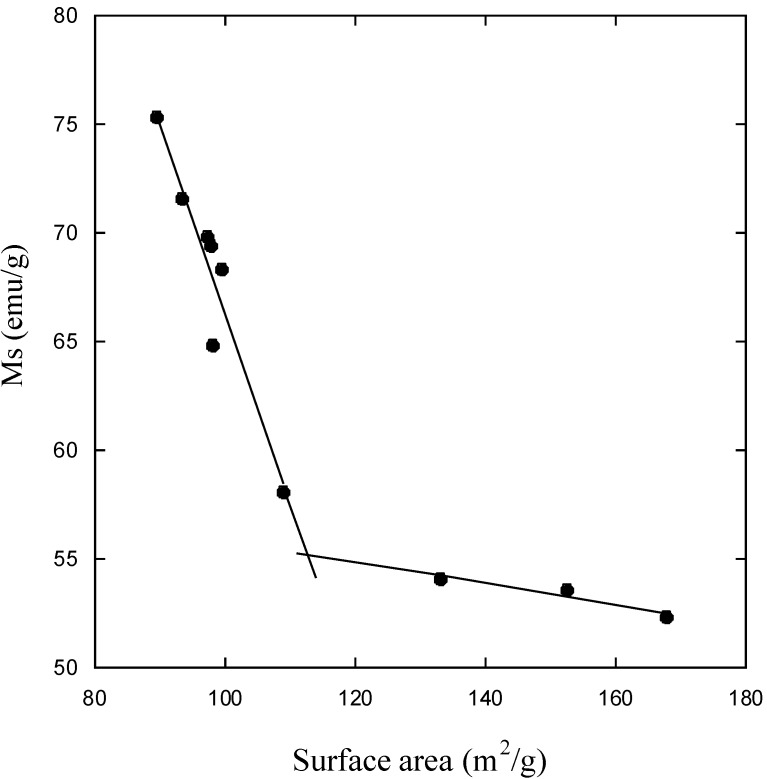
Saturation magnetization (Ms) as a function of magnetite surface area.

The nanosized magnetite samples display superparamagnetism and saturation magnetization (Ms) values smaller than the bulk magnetite value, 92 emu/g. On the other hand, the synthesized samples show large differences regarding the Ms values measured at room temperature (Table 4). The reduction of Ms can be prevalently related with the magnetite size. A linear correlation between Ms and the particle size of magnetite results, as can be seen in Figure 9 for particle sizes ranging between 9 and 12 nm. An analogous correlation results between Ms and surface area (Figure 10). This linear correlation between Ms and particle size was reported for maghemite [42]. A second linear correlation with a lower gradient can be seen in the 6.5–8.0 nm size range of magnetite particles.

Several explanations have been provided on the decrease of the Ms with the reduction of the particle size of magnetite. One factor concerns the entity of the spin disorder layer, which increases with a decrease in crystallite size [33]. Another factor for the reduction of the magnetic moment can be also explained through the effect of a dipolar interaction between magnetite nanoparticles [32]. The irregular morphology of magnetite particles might influence the value of Ms as a contribution from surface anisotropy [34]. As all the synthesized samples are almost spherical in shape, a zero contribution from surface anisotropy must be expected [43]. A further reduction of Ms could be attributed to incomplete crystallization of magnetite after the reaction synthesis. The decrease in Ms can be also due to changes in A and B site population [44]

The presence of a bend in Figure 9 involves a different trend of Ms as a function of magnetite size. A possible explanation could be related to the nature of the countercation of the base, which affects the degree of agglomeration: *i.e.*, the decrease of magnetite particle size determines a decrease of the agglomeration in the 6.5–8.0 nm size range, so contrasting with the reduction of Ms.

## 5. Conclusions

A cheap co-precipitation method has been utilized to synthesize magnetite nanoparticles exhibiting superparamagnetic behavior. They were formed by a one pot co-precipitation reaction in a large pH window (10.0–13.0) at room temperature. The size reduction of magnetite nanoparticles, precipitated with a certain base, is affected by both the pH and the slow or fast addition of the basic solution to the solution of mixed Fe^2+^ and Fe^3+^ ions. A further reduction of magnetite size can be determined by the nature of the precipitating base in accordance with the following sequence: (C_2_H_5_)_4_NOH < KOH < NaOH.

The resulting magnetite nanoparticles exhibit superparamagnetic properties, depending on the particle size: the lower the particle size, the lower is the saturation magnetization (Ms).

The agglomeration among the magnetite nanoparticles determines the formation of mesopores, whose average size is affected by the size of individual particles. Mesopores larger in size result, in fact, in bigger particles, whereas mesopores smaller in size result in smaller particles. The degree of agglomeration, determined by the interface area among the individual particles, affects the Ms value. Its reduction with the decrease of magnetite particle size becomes less marked for less agglomerated particles: *i.e.*, magnetite synthesized in the presence of a base with a counteraction bigger in size (C_2_H_5_)_4_NOH.

The synthesized magnetite with different mesoporous structures appears very promising for biomedical applications. It is well known that because of the low drug or enzyme loading on the conventional magnetite nanoparticles, the magnetic material is often incorporated into mesoporous materials to form a hybrid support with a consequent reduction of Ms. The synthesized products, being combinations of mesoporous properties with a magnetic property, appear to be of interest for biomedical applications.
